# The Use of Ergot in Post-Partum Hemorrhage

**Published:** 1849

**Authors:** 


ARTICLE VI.—*The use of Ergot in Post-Partum Hemorrhage.*

We have mentioned the administration of ergot of rye as a preventive of post-partum hemorrhage; and in the hospital we have seen such decidedly favorable results from its use, when employed for this purpose, as to have no hesitation in pronouncing the practice to be both safe and efficient. With this intention it may be given at one or other of three periods: namely, when the head of the child is on the perineum, and about to be expelled; or immediately after the head has cleared the os externum, and before the shoulders have passed; or, thirdly, so soon as the insertion of the cord into the placenta can be felt. "By giving ergot before the child has been expelled, some time may be gained; but should the placenta be morbidly adhering to the uterus, the dfficulty of introducing the hand for its removal will be greatly increased. By adopting the third plan, this source of apprehension is avoided. To this method it may be objected that much time will, perhaps, elapse, and a considerable quantity of blood be lost, before the ergot is administered; nevertheless, the possibility of the placenta being morbidly adherent, should be ever present in the mind of the practitioner, and deter him from resorting to a measure which may so greatly augment the danger of the complication." Dr. Johnson, who introduced the practice into this hospital, generally gives the ergot according to the mode last recommended. In certain instances, however, where from previous losses it was a matter of the utmost importance to prevent any further hemorrhage after delivery, we have not scrupled to administer it in the second way spoken of above, and hitherto without any unpleasant effect. Here, as on every other occasion, we should be careful to use ergot of undoubted genuine quality, for otherwise its exhibition can be productive of no good, and will only cause disappointment. Few medicines so readily spoil, or are to be found of such variable quality; and this circumstance goes far, we think, to reconcile the conflicting opinions which have been entertained respecting its properties and doses.—*M'Clintock and Hardy's Practical Observations*, pp. 220 and 221, *Ibid.*

